# An experimental hut evaluation of Olyset^® ^nets against anopheline mosquitoes after seven years use in Tanzanian villages

**DOI:** 10.1186/1475-2875-7-38

**Published:** 2008-02-28

**Authors:** Robert C Malima, Stephen M Magesa, Patrick K Tungu, Victor Mwingira, Frank S Magogo, Wema Sudi, Frank W Mosha, Christopher F Curtis, Caroline Maxwell, Mark Rowland

**Affiliations:** 1 National Institute for Medical Research, Amani Medical Research Centre, P.O. Box 81, Muheza, Tanzania; 2 Kilimanjaro Christian Medical Centre, P.O. Box 2228, Moshi, Tanzania; 3 London School of Hygiene and Tropical Medicine, WC1E 7HT, London, UK

## Abstract

**Background:**

Long-lasting insecticidal nets (LLINs) are advocated by WHO for protection against malaria. Of the three brands of LLINs currently approved by WHO, Olyset^® ^is the only one currently granted full recommendation. With this type of LLIN, the insecticide (permethrin) is incorporated into the polyethylene fibre during manufacture and diffuses from the core to the surface, thereby maintaining surface concentrations. It has not been determined for how long Olyset nets remain protective against mosquitoes in household use.

**Methods:**

Examples of Olyset nets, which had been in use in Tanzanian villages for seven years, were tested in experimental huts against naturally entering *Anopheles gambiae *and *Anopheles funestus *mosquitoes. Performance was compared with new Olyset nets, conventionally treated ITNs (either newly treated with alphacypermethrin or taken from local villages after 1.5 years of use) and untreated nets. All nets were artificially holed except for the seven-year Olyset nets, which had developed holes during prolonged domestic use.

**Results:**

*Anopheles funestus *and *An. gambiae *in NE Tanzania are susceptible to pyrethroids. The new Olyset nets caused high mortality against *An. funestus *(73.9%) and *An. gambiae *(62.7%) in experimental huts. The seven-year Olyset nets caused 58.9% mortality against *An. funestus *and 40.0% mortality against *An. gambiae*. The freshly treated alphacypermethrin nets also caused high mortality against *An. funestus *(70.6%) and *An. gambiae *(72.0%); this decreased to 58.4% and 69.6% respectively after 1.5 years of use. The new Olyset nets inhibited blood-feeding by 40–50%. The 7 year Olyset nets showed no feeding inhibition over that shown by the untreated nets. The alphacypermethrin treated nets failed to inhibit blood-feeding after 1.5 years of use. However iHhhdn laboratory tunnel tests samples of all types of treated net including the 7 year Olyset inhibited blood-feeding by more than 95%.

**Conclusion:**

After seven years of use Olyset nets were still strongly insecticidal. Mosquito mortality decreased by only 20–35% over this period. However, Olyset would not provide personal protection after seven years unless it was in good condition and all holes fully repaired.

## Background

Long-lasting insecticidal nets (LLINs) in which the insecticide treatment is intended to last for the lifetime of the net are advocated by the World Health Organization for protection against malaria [1]. LLINs have in recent years come to dominate the institutional net-buying market [2,3]. Of the 3 brands of LLIN currently approved by the WHO Pesticide Evaluation Scheme, Olyset^® ^(Sumitomo Chemical Co., Ltd, Japan) is the longest established and is the only LLIN to be granted full, as opposed to interim, recommendation [3,4]. With this brand of LLIN, the pyrethroid insecticide (permethrin) is incorporated into the polyethylene fibre during manufacture and diffuses gradually from the core to the surface. Surface concentrations are regenerated after removal of permethrin by washing or general use [4-6]. Olyset nets are manufactured with a 4 mm mesh which is considerably larger than the 1.5 mm of most bed nets; the larger mesh has the advantage of allowing more ventilation for the sleeper. Olyset has been in use in some Tanzanian villages for several years and according to cone bioassay tests retains insecticidal activity for at least seven years [7].

In contrast to this finding, a study of Olyset carried out after two years of household use produced bioassay mortality rates that fell below the threshold set by WHOPES for LLIN [8]. Simple cone bioassay tests in which mosquitoes are exposed to treated netting in plastic containers for a few minutes do not reliably measure operational effectiveness, and interpretation of mortality data is confounded when the insecticide under test shows repellent as well as toxic activity [9]. Permethrin is well known for its excito-repellent activity [10]. Probably the best way to estimate operational effectiveness of LLIN after an interval in the field is to retrieve the nets from householders and to re-evaluate the nets under the realistic but controlled conditions offered by experimental huts since this technique is able to measure in a realistic way what proportion of mosquitoes are inhibited from entering the room with a person sleeping in the net, the proportion of mosquitoes inhibited from biting and feeding, and the proportion killed by the net. In experimental hut trials, volunteers take it in turns to sleep under the nets in the presence of host-seeking mosquitoes, alternating the test net with new LLIN/ITN (positive control) or untreated nets (negative control), and in this way relative protectiveness is demonstrated. In the study reported here some of the Olyset nets reported in Tami et al [7], which had been in use for seven years in Tanzanian villages, were tested in experimental huts against naturally entering *Anopheles gambiae *and *Anopheles funestus *mosquitoes. The effectiveness of these Olyset nets was examined relative to that of new Olyset nets, untreated control nets and conventional ITNs treated with alphacypermethrin (either newly treated or taken from local villages after 1.5 years of use).

## Materials and Methods

### Study area and experimental huts

Four veranda trap huts were constructed according to a basic design first described by Smith [11] with substitution of concrete for wooden floors. Surrounding each of the huts is a 10 cm wide moat filled with water to prevent scavenging ants from entering. The working principle of these huts has been described by Smith & Webley [12] and Curtis *et al *[13]. The huts are situated at Zeneti village in Muheza district, north-east Tanzania (5°13'S and 38°39'E, altitude 193 m). *Anopheles gambiae *s.s. and *An. funestus *are the predominant mosquito species in the area [14].*Anopheles funestus *is responsible for most of the dry season transmission of *Plasmodium falciparum*, and *An. gambiae *s.s. is responsible for wet season transmission.

### Bed nets and treatments

The Olyset nets were formerly distributed in the villages of Mbwawa, Kibaha district [15] and Mvumi, Dodoma district [16] in 1994–1995, and were retrieved in July 2002 for laboratory evaluation by Tami *et al *[7]. Two of the nets were stored in plastic bags at room temperature until June 2005 and then tested in experimental huts at Zeneti. The new Olyset nets were obtained from Sumitomo Chemicals.

The alphacypermethrin nets were treated at a rate of 20 mg/m^2 ^and distributed to houses in Kilulu village near Muheza in October 2003. No re-treatment was carried out. Five nets were retrieved in June 2005 after 20 months of use. The new alphacypermethrin nets were treated at a rate of 20 mg/m^2^.

Nets had six 4 cm diameter holes cut on sides and ends to simulate the condition of worn or torn nets. The 7 year Olyset already had a comparable number of holes as a result of prolonged domestic use. Five nets per treatment arm were prepared for testing in the huts except that only two seven-year Olyset nets were available for use in huts.

### Experimental hut evaluation

The evaluation was run over 60 days between 20 June and 3 September 2005. The five treatments (including the untreated control net) were rotated through each of the four huts twice using a Latin square design. A treatment was allocated to a hut for five consecutive nights before being rotated to another hut. A different net was used on each of the five nights of the rotation. With five treatments and four huts, it was necessary for 1 treatment to drop out of the evaluation during each rotation. Two volunteers from Zeneti village slept in each hut between 19:30 and 6:30 hours. Sleepers were rotated between huts on successive nights in order to reduce the effect of variation in individual attractiveness to mosquitoes. Likewise, the direction of the two open verandas was routinely exchanged with the treatment rotation in order to minimise the potential confounding factor of preferential escape in one or other direction. Huts were cleaned and left to air for at least one night between each five day rotation.

Mosquitoes were collected in the morning at 07:00 from inside the net, the window (exit) traps as well as from the ceiling, walls and floor of the veranda and inside the room. The collected mosquitoes were kept in paper cups and brought to the field laboratory for species identification, determination of gonotrophic condition, and mortality counts. All live mosquitoes were held in paper cups supplied with 10% glucose solution and held for 24 hours after which delayed mortality was scored.

### Analysis

The numbers of mosquitoes in the two verandah traps were multiplied by two to adjust for the unrecorded escapes through the other two verandahs which are left unscreened to allow routes for entry of wild mosquitoes via the gaps under the eaves [11-13].

The data was double entered and analysed to show the effect of each treatment in terms of:

• Insecticide-induced exiting: percentage of the total mosquito collection from verandah and exit traps in a treatment hut relative to the control.

• Blood feeding inhibition: percentage of unfed mosquitoes from a treatment hut relative to the control.

• Overall mortality: total number of mosquitoes found dead 24 hours after collection.

Assessment of outcome variables between treatments relative to the control was analysed using logistic regression for proportional data and non-parametric tests for numeric data using STATA^® ^statistical analysis software package version 8 (Stata corporation, Collage Station TX, USA, 2003).

### Contact bioassays

Before subjecting the treated nets to experimental hut evaluation, contact bioassays were carried out according to WHOPES guidelines [17]. Susceptible laboratory-reared *An. gambiae *(Kisumu strain) were exposed in batches of five to treated or untreated netting in WHO cones for three minutes, after which they were held for 24 hours for mortality scoring.

### Tunnel tests

These were carried out in apparatus designed to simulate experimental hut conditions [18]. The tunnel is a glass cuboid measuring 60 cm long, 25 cm high and 25 cm wide, with three chambers (release, middle and baited). The test netting sample has nine evenly spaced 1 cm diameter holes and is fixed on a cardboard frame and placed at the separation between the middle and the baited (guinea pig) chamber. The partition between release and middle chamber has a single 10 cm diameter hole which is intended to prevent mosquitoes contacting the treated netting before starting bait-seeking flights.

Test mosquitoes were *c*. 100 non-blood fed, 4–6 days old, insectary-reared *An. gambiae *(Kisumu strain) or *Culex quinquefasciatus *(Masimbani strain). These were introduced into the releasing chamber of the tunnel at 18:00 and recovered at 06:00 the next morning. The cage was maintained at 26°C and 80% relative humidity. In the morning mosquitoes were removed and numbers scored separately from each chamber for estimation of penetration, blood feeding and mortality rates. Mosquitoes were held with sugar solution for a further 24 h before scoring delayed mortality.

### Resistance tests

Samples of adult *An. gambiae *and *An. funestus *collected from the experimental hut site were tested for resistance status on permethrin 0.75% or deltamethrin 0.05% papers in WHO test kits according to WHOPES guidelines (17). Time taken for 50% and 90% knockdown of mosquitoes (KDT_50 _and KDT_90_) and 95% confidence intervals were determined by probit analysis using the computer program PoloPlus (Version1.0, LeOra Software).

### Ethical clearance

Informed consent was obtained from all volunteers recruited to the experimental hut study. The study was approved by ethics committees of LSHTM and NIMR (Ref: NIMR/HQ/R.8a/Vol. X/86).

## Results

### Resistance tests

Wild caught *An. gambiae *and *An. funestus *from Zeneti village were fully susceptible to permethrin 0.75% and deltamethrin 0.05% test papers (Table 1).

**Table 1:** Susceptibility of wild Anopheles from Zeneti village to Permethrin and Deltamethrin

Species	Insecticide treated papers	No. of mosquitoes tested (n)	24 h Mortality	KDT_50 _(95% CI)	KDT_90 _(95% CI)
*An. gambiae*					
	Permethrin 0.75%	164	100	10.5 (5.97–13.22)	24.6 (19.3–44.7)
	Deltamethrin 0.05%	166	100	12.03 (10.7–13.1)	20.9 (18.8–24.3)
*An. funestus*					
	Permethrin 0.75%	229	100	10.23 (8.7–11.3)	15.8 (14.1–19.2)
	Deltamethrin 0.05%	104	100	11.9 (8.9–14.0)	19.8 (16.5–31.0)

KDT_50 _= Time for 50% knockdown in minutes

KDT_90 _= Time for 90% knockdown in minutes

95%CI = 95%Confidence Interval

### Experimental hut trial

The total number of mosquitoes collected during 60 nights was 1,108, consisting of *An. gambiae *(32.1%), *An. funestus *(59.5%) and *Cx. quinquefasciatus *(8.4%). The mean number caught per night was 18.5, consisting of 17 *Anopheles *and 1.5 *Culex *mosquitoes. A summary of results for *An. gambiae *and *An. funestus *is shown in Tables 2 and 3. *Cx. quinquefasciatus *were too few in number to provide meaningful conclusions.

**Table 2:** Comparison of Olyset and alphacypermethrin treated nets against *Anopheles gambiae *in experimental huts.

	**Untreated net**	**New alphacypermethrin**	**1.5 year alphacypermethrin**	**New Olyset**	**7 year Olyset**
Total females caught	**112**	**93**	**138**	**177**	**165**
Females caught/night	1.9^a^	1.5^a^	2.3^a^	2.9^a^	2.7^a^
% Inside net	8.9^a^	1.1^b^	0.7^b^	0^b^	0^b^
% In veranda and exit traps (95% C.I.)	91.1^a ^(85.8 – 96.4)	94.6^ab ^(90.0 – 99.2)	97.1^b ^(94.3 – 99.9)	98.3^b ^(96.4 – 100.2)	97.0^b ^(94.5 – 99.6)
% Insecticide induced exophily	-	39.3	67.5	81.0	66.1
% Blood fed (95% C.I.)	26.8^a ^(18.6 – 35.0)	8.6^b ^(2.9 – 14.3)	18.8^ab ^(12.3 – 25.4)	15.8^b ^(10.4 – 21.2)	49.5^c ^(40.9 – 56.1)
% Blood feeding inhibition	-	67.9	29.9	40.9	0
% Mortality (95% C.I.)	3.6^a ^(0.13 – 7.0)	72.0^bc ^(62.9 – 81.2)	69.6^c ^(61.9 – 77.2)	62.7^b^(55.8 – 69.8)	40.0^d ^(32.5 – 47.5)
% Mortality (corrected for control)	-	71.0	68.4	61.3	37.9

Numbers in the same row sharing a letter superscript do not differ significantly (*P *> 0.05)

**Table 3:** Comparison of Olyset and alphacypermethrin treated nets against *Anopheles funestus *in experimental huts.

	**Untreated net**	**New alphacypermethrin**	**1.5 year alphacypermethrin**	**New Olyset**	**7 year Olyset**
Total females caught	**315**	**211**	**291**	**222**	**214**
Females caught/night	5.2^a^	3.5^b^	4.8^ab^	3.7^b^	3.6^b^
% Inside net	5.7	0	1.4	0.5	0.9
% In veranda and exit traps (95% C.I.)	91.4^a ^(88.3 – 94.5)	99.5^b ^(98.6 – 100.4)	96.2^c ^(94.0 – 98.4)	92.8^ac ^(89.4 – 96.2)	95.3^ac ^(92.5 – 98.2)
% Insecticide induced exophily	-	94.2	55.8	16.3	45.3
% Blood fed (95% C.I.)	32.4^a ^(27.2 – 37.6)	10.0^b ^(5.9 – 14.0)	29.2^a ^(24.0 – 34.4)	16.2^b ^(11.4 – 21.1)	36.0^a ^(30.0 – 42.4)
% Blood feeding inhibition	-	69.3	9.9	49.9	0
% Mortality (95% C.I.)	7.9^a ^(4.9 – 10.9)	70.6^bd ^(64.5 – 76.8)	58.4^c ^(52.8 – 64.1)	73.9^b ^(68.1 – 79.7)	58.9^cd ^(52.3 – 65.5)
% Mortality (corrected for control)	-	68.1	54.8	71.6	55.3

Numbers in the same row sharing a letter superscript do not differ significantly (*P *> 0.05)

In experimental hut trials, where all treatments are presented simultaneously, comparison between the mean numbers collected from untreated control and treated huts are used to give estimates of the deterrent or spatial repellent effects of pyrethroid treatments. In the present trial where the number of treatments exceeded the number of huts available, these estimates could not be reliably made because treatments took it in turn to drop out and there were changes in the population of mosquitoes entering the huts during the course of the trial.

### Exiting rates

The overnight exiting rates of *An. gambiae *ranged from 91.1% in the untreated control to 98.3% in the huts with the new Olyset nets. Tables 2 and 3 show estimates of the proportions induced to exit by the insecticide relative to those which would have exited without insecticide stimulation. All insecticide treatments led to higher exiting rates and this was statistically significant for Olyset and 1.5 year alphacypermethrin treatments against *An. gambiae*. The overnight exiting rate of *An. funestus *from the control huts was similar to that shown by *An. gambiae*. Only the alphacypermethrin treatments induced exiting rates of *An. funestus *that were significantly greater that the control.

### Blood feeding

The blood feeding rates through holed, untreated netting were *c*.30% in both *An. gambiae *and *An. funestus*. Insecticide-induced inhibition of blood feeding ranged between 40% and 50% in the presence of new Olyset nets and between 68% and 72% in the presence of new alphacypermethrin treated nets. The blood-feeding rates through the household-used alphacypermethrin nets were less than through the untreated nets, but the differences were not significant. There was no reduction in blood feeding through the seven-year Olyset nets relative to the untreated nets. Very few mosquitoes were collected from inside any of the treated nets indicating that few penetrated the holes or through the larger mesh of the Olyset nets.

### Mortality

The new alphacypermethrin nets induced high rates of mortality against *An. gambiae *and *An. funestus*. A difference in mortality rate between the new and the 1.5 year alphacypermethrin treatments was evident in the *An. funestus *results but not in the *An. gambiae *results. The new Olyset also scored high mortality. After seven years of use the Olyset nets were still very insecticidal; mortality of *An. funestus *was only 20% lower and mortality of *An. gambiae *was 36% lower relative to the new Olyset nets.

The local *Cx. quinquefasciatus *are highly resistant to pyrethroids, and too few were collected in the huts (100 mosquitoes in 60 nights) to draw major conclusions. Only 33% (7/21) were killed by the new Olyset nets and only 15% (3/20) were killed by the seven-year Olyset. The mortality rates with alphacypermethrin were 25% (4/16) for the new treatment and 6.9% (2/29) for the 1.5 year treatment.

### Cone bioassays

*Anopheles gambiae *(KISUMU) showed knockdown rates ranging from 56%–88% and mortality rates ranging from 84–99% after 3 min exposure to samples of treated netting (Table 4). Exposure to the 7 year Olyset induced a mortality rate (89%) similar to that induced by the fresh alphacypermethrin treatment. The locally collected *Cx. quinquefasciatus *Masimbani strain is highly resistant to pyrethroids and few mosquitoes were killed by any of the treatments, whether old or newly treated.

**Table 4:** Summary of cone bioassay tests on nets collected from the field. 100 adult females were tested on each treatment.

	Untreated net	New alphacypermethrin	1.5 year alphacypermethrin	New Olyset	7 year Olyset
*An. gambiae *Kisumu strain					
% Knockdown at 60 min	0^a^	88^b^	70^c^	75^c^	56^d^
% Mortality after 24 hours	0^a^	84^b^	87^b^	99^c^	89^b^
*Cx. quinquefasciatus *Masimbani strain					
% Knockdown at 60 min	1^a^	1^a^	0^a^	5^a^	0^a^
% Mortality after 24 hours	0^a^	2^ab^	9^cd^	17^c^	7^bd^

Numbers in the same row sharing a letter superscript do not differ significantly (*P *> 0.05)

### Tunnel tests

All treatments reduced the numbers of *An. gambiae *penetrating the holed netting (Table 5). A greater proportion of *An. gambiae *penetrated the seven-year Olyset netting than penetrated the new Olyset netting. Passage of the pyrethroid resistant *Cx. quinquefasciatus *through the netting was less inhibited by the insecticide treatments than was the passage of susceptible *An. gambiae*. Generally, the permethrin in Olyset was more inhibitory to passage than the alphacypermethrin treatments were.

**Table 5:** Summary of tunnel test studies on the five types of net.

	Untreated net	New alphacypermethrin	1.5 year alphacypermethrin	New Olyset	7 year Olyset
*An. gambiae *Kisumu strain					
Total tested	103	122	103	102	99
% Passage (95% C.I.)	99^a ^(94.7–99.9)	41^b ^(32.1–50.2)	15.5^c ^(9.1–23.9)	27.5^d ^(19.1–37.1)	56.6^e ^(46.2–66.5)
% Passage inhibition	-	59	84	72	43
% Feeding (95% C.I.)	83.5^a ^(74.8–90)	1.6^b ^(0.1–5.7)	1.9^b ^(0.2–6.8)	2.9^b ^(0.6–8.3)	0^b ^(0.0–3.7)
% Feeding inhibition	-	98	98	97	100
% Mortality (95% C.I.)	7.8^a ^(3.4–14.7)	96.7^b ^(91.8–99.1)	81.6^c ^(72.7–88.5)	95.1^b ^(88.9–98.3)^b^	76.8^c ^(67.2–84.6)

*Cx. quinquefasciatus *Masimbani strain					
Total tested	118	118	109	102	104
% Passage (95% C.I.)	78^a ^(69.4–85)	68.6^ab ^(59.5–76.8)	76.1^a ^(67–83.8)	47.1^c ^(37–57.2)	55.8^bc ^(45.6–65.5)
% Passage inhibition	-	12	2	40	28
% Feeding (95% C.I.)	73.7^a ^(64.8–81.4)	61.9^ab ^(52.4–70.7)	74.3^a ^(65–82.2)	37.3^c ^(27.8–47.3)	46.2^bc ^(36.3–56.2)
% Feeding inhibition	-	16	0	49	37
% Mortality (95% C.I.)	0^b ^(0.0–3)	3.4^b ^(0.9–8.4)	0.9^b ^(0–5)	9.8^b ^(4.8–17.2)	5.8^b ^(2.1–12.2)

Numbers in the same row sharing a letter superscript do not differ significantly (*P *> 0.05).

Blood-feeding in *An. gambiae *was almost fully inhibited by the Olyset and alphacypermethrin treatments. Blood-feeding rates in *Cx. quinquefasciatus *were inhibited more by the permethrin of Olyset than by alphacypermethrin; feeding rates were higher in tests with the older treatments.

More than 95% *An. gambiae *were killed by the new Olyset and new alphacypermethrin treatments. Significantly smaller proportions were killed by the treatments which had been used in households; nevertheless 56.6% *An. gambiae *did succumb to the seven-year Olyset treatment. Very few of the pyrethroid resistant *Cx. quinquefasciatus *were killed by either alphacypermethrin or Olyset.

## Discussion

The Olyset nets acquired from the Tanzanian villages after seven years of household use had in the earlier bioassay tests conducted by Tami *et al *(7) produced results (92% knockdown and 50% mortality) of similar magnitude to the bioassay tests presented here. Tami *et al *[7] demonstrated that the nets retained 30–40% of the original insecticide content after seven years of household use. The relationship between laboratory bioassay results and field efficacy of nets under household conditions is poorly understood [19,20]. It is not possible to make predictions from bioassay data as to whether the observed insecticidal effect is sufficient to kill mosquitoes or protect against biting in a household or community setting. Only field studies can provide an unequivocal answer, and experimental hut trials are a controlled and objective way to obtain such information. Randomised controlled trials measuring changes in malaria incidence constitute the gold standard for evaluating treated nets but while it would be feasible to run such trials with new LLIN, attempting a controlled trial with 7y old nets or interpreting results 7y after the introduction of new LLINs would be problematic to say the least. Experimental hut trials measuring personal protection or insecticide-induced mosquito mortality are simple, comparatively cheap to undertake and give clear answers about current effectiveness of older nets. The conclusion is that after seven years of household use the Olyset nets had lost only a third of their efficacy against *An. gambiae *and *An. funestus*. These results are consistent with those obtained in experimental huts with Olyset net obtained from other communities after three [4] and four years of use [21].

A possible limitation of experimental hut trials is that only a handful of nets can be tested at any one time; WHO recommends testing 5 nets per treatment arm [18]. In the present trial only two 7y Olyset were available for testing but the results for each was consistent with the other and this raises confidence in the results.

This study was carried out in an area of NE Tanzania where *An. gambiae *and *An. funestus *are fully susceptible to pyrethroids and *Cx. quinquefasciatus *is highly resistant [22]. The trial coincided with the end of the rainy season and the start of the dry season when there is a natural succession from *An. gambiae *to *An. funestus*. *Cx. quinquefasciatus *were not abundant at this time but it was still clear, even with the low numbers collected, that the pyrethroid treated nets had little impact on this species in the field (or in laboratory cone or tunnel tests) owing to the resistance expressed in the *Culex *populations around Muheza [22]. In the present trial, five treatment arms were being tested in four experimental huts and it was therefore necessary to systematically drop each treatment during the course of the rotations. Unfortunately, owing to variation in weekly mosquito abundance it was difficult to estimate with accuracy the existence of spatial repellency (deterrence) since this calculation requires comparison of the number of mosquitoes captured in rooms with different treatments rather than the proportion of mosquitoes affected by a treatment within a particular room (in contrast to the estimates of the proportion of mosquitoes prevented from blood-feeding or the proportion killed). With the benefit of hindsight it is better to reduce the number of treatments or increase the number of huts to match the number of treatments than have individual treatments phase in or out of the trial.

The experimental huts showed that, when new, both alphacypermethrin and Olyset nets provide significant protection against mosquito bites. However, after 1.5 years of use in the case of alphacypermethrin and seven years in the case of Olyset the nets failed to prevent mosquito biting despite still killing many *An. gambiae *and *An. funestus*. This observation is not consistent with the results of tunnel tests which showed that the seven-year Olyset net provided a level of protection and blood feeding inhibition similar to the other treatments. Trials by Maxwell *et al*. with 4y old nets in experimental huts found quite a good reduction in biting [21]. It seems probable that tunnel tests overestimate the field effectiveness of treatments compared to hut trial estimates. In hut trials parts of the sleepers' bodies may touch the sides of the net giving mosquitoes an opportunity to feed through the netting. Olyset net is highly resilient to wear-and-tear but even Olysets can develop holes after seven years, and the comparatively large holes that had developed probably contributed to the high rate of blood feeding. Regular maintenance and repair of holes would help restore protection against mosquito biting, making the nets better tools for personal protection. Extrapolating from the mortality results, it is predicted that were Olysets to be used by the majority of households in the community the overall impact on mosquito population density and longevity should be sufficient to achieve considerable reduction in malaria transmission even after seven years of use. To prevent biting of users, programmes may find it necessary to replace Olyset nets after about 7y. Further studies of the persistence of effectiveness under village conditions are urgently needed.

## Conclusion

After seven years of regular use, the LLIN Olyset remained highly insecticidal to mosquitoes that came into contact with it under field conditions. Personal protection, as measured by a reduction in blood-feeding rate relative to that of untreated nets, was evident in laboratory tunnel tests but not under field conditions. Personal protection would require all holes to be mended and for occupants to not touch the sides of the netting while sleeping. Further studies on the persistence of effects of LLINs under village conditions are urgently needed.

## Abbreviations

LLIN Long-lasting insecticidal net,

WHO World Health Organization,

ITN Insecticide-treated net,

WHOPES World Health Organization Pesticide Evaluation Scheme,

LSHTM London School of Hygiene & Tropical Medicine,

NIMR National Institute of Medical Research,

KCMC Kilimanjaro Christian Medical Centre.

## Authors' contributions

RCM supervised the project, carried out the analysis and drafted the manuscript.

SMM managed the project and entomological teams, participated in the design of the study and contributed to drafting the manuscript.

PTT, VM, FSM and WS carried out trial, processed the data and helped to analyse the data.

FWM and CFC contributed to the study design and critically reviewed the manuscript.

CM contributed to the study design and supervision of research, and critically reviewed the manuscript.

MR contributed to study design and data analysis and revised the manuscript.

All authors read and approved the final manuscript.
